# A preliminary study of cross-amplified microsatellite loci using molted feathers from a near-threatened Painted Stork (*Mycteria leucocephala*) population of north India as a DNA source

**DOI:** 10.1186/s13104-017-2932-y

**Published:** 2017-11-21

**Authors:** Bharat Bhushan Sharma, Basu Dev Banerjee, Abdul Jamil Urfi

**Affiliations:** 1 Department of Environmental Studies, University of Delhi, Delhi, 110007 India; 2 Environmental Biochemistry & Molecular Biology Laboratory, Department of Biochemistry, University College of Medical Sciences & Guru Teg Bahadur Hospital, University of Delhi, Dilshad Garden, Delhi, 110095 India

**Keywords:** Painted Stork, Molted feathers, Microsatellites, Wood Stork, Cross-amplification, Genetic diversity, India

## Abstract

**Objective:**

In continuation of an earlier study in which we reported the cross-amplification of Wood stork microsatellites on the DNA obtained from molted feathers of Painted stork (*Mycteria leucocephala*), here we investigated the nature of cross-amplified microsatellites and the effect of non-invasive samples on cross-amplification success. In a limited manner, we also addressed the genetic diversity and differentiation in a north Indian population of the Painted Stork examined over three nesting seasons.

**Results:**

Among the nine cross-amplified loci, only 5 were polymorphic. Three and 6 loci exhibited low (< 50%) and high amplification success rates (> 80), respectively. For 36 of 145 samples most of the loci failed to amplify. For genetic diversity, only 3 loci could be used since others exhibited low amplification and linkage disequilibrium. Probability of identity (0.034) was not low enough to develop a confidence that the similar genotypes originate from the same individual. Forty-two unique genotypes were identified. In 3 loci, a low to moderate level of genetic diversity (mean He = 0.435) was reported. Non-significant Fst (0.003, P = 0.230), G’stH (0.005, P = 0.247) and Dest (0.003, P = 0.250) values indicate a lack of structuring in temporally distributed populations of Delhi Zoo. The limitations and uniqueness of this study are discussed.

## Introduction

The near threatened Painted Stork (PS) (*Mycteria leucocephala*) is widespread across south and south-east Asia with a stronghold in India. While in the non-breeding season these waterbirds remain widely dispersed across the countryside, after the cessation of the summer monsoon showers they start congregating at their traditional nesting sites which are trees planted on islands in village tanks or in urban premises. One important nesting site in north India is located within the confines of the Delhi Zoological Park in India’s capital city. A wild PS population established itself in 1959–1960 and has been regularly visiting the park for nesting since its introduction [1]. No previous genetic studies of the PS have been conducted in India, yet such studies are key to understating geographic patterns of genetic diversity of these storks in the Indian subcontinent and devising conservation strategies [2].

In continuation of our earlier work [3] in which we reported the successful cross-amplification of Wood Stork (WS) (*Mycteria americana*) microsatellites on PS DNA obtained exclusively from molted feathers, here we investigate the nature of cross-amplified loci and the effect of non-invasive samples on cross-amplification success of different loci. In addition, we report results of estimates of genetic diversity obtained across three nesting seasons.

## Main text

### Materials and methods

The present study was conducted by using DNA extracted from molted feathers obtained from the PS nesting colony in the premises of the National Zoological Park (Delhi Zoo), Delhi (28° 36′ 21.11″ N, 77° 14′ 48.47″ E). One hundred and forty-five feather samples (142 molted and 2 plucked from dead individuals) were collected over a 3 year period: (1) 2011–2012—47 samples, (2) 2012–2013—45 samples and (3) 2013–2014—53 samples.

For DNA extraction, barbs of the vane were removed and the calamus region was cut and washed in 70% ethanol for 30 min followed by a 30 min wash in ddH_2_O. The basal tip of the calamus and the blood clot from the superior umbilicus [4] were put in a microcentrifuge tube and finely chopped with sterile scissors. The details of the standardized isopropanol method of DNA extraction [5] have already been described in Sharma et al. [3].

Nine Wood Stork microsatellites i.e. WSµ03, WSµ08, WSµ09, WSµ13, WSµ17, WSµ18, WSµ20, WSµ23 and WSµ24 (Table 1) [6] were subjected to amplification of the extracted DNA. Reaction conditions for the polymerase chain reaction (PCR) included the following: 1× buffer, 2.5 mM MgCl_2_, 0.2 mM dNTPs, 1.25 U Taq DNA polymerase (AmpliTaq Gold, Thermo Fisher Scientific, USA), 0.8 mg/ml BSA, 0.40 µM primers and 60–100 ng DNA template. Cycling conditions were as follows: initial denaturation at 95 °C for 8 min, followed by 40 cycles of 30 s denaturation at 96 °C, 30 s annealing at specific annealing temperature [3] and 75 s extension at 72 °C. A final extension was performed at 72 °C followed by a hold at 4 °C. PCR products were visualized and sized on an ABI 3130xl Genetic Analyzer (Applied Biosystems).

**Table 1:** Allele sizes and amplification success rate of the microsatellite loci used in this study

Locus	Primer sequence (5′–3′)	Number of alleles	Allele size range (bp)	Amplification success (%)
WSµ03	F-AGAAGCCAAATTGATTAGA R-ACAAAGTTGCGGAGAA	1	166	92.75
WSµ08	F-TGTCTTTCCAGGTAGTTTT R-TACAACTGTTCGTGCTTT	1	180	43.48
WSµ09^a^	F-GGTAACAGCGAGTTGGAT R-TAATGCCAATAAGTGCTTAG	2	270–273	90.83
WSµ13	F-AGGGCTCATCAATAGTGT R-GTTTGCCCACTGTGTCAACT	5	222–230	24.64
WSµ17^a^	F-GGCAAGCTGTTATACTAAT R-GTTTTTCATATACTAACTGG	4	236–246	80.73
WSµ18^a^	F-CATATACTAACTGGGTTTAATC R-GTTGTTCTGCGTTATTC	4	277–287	86.4
WSµ20	F-CGGGCCTTTATCTATC R-ACAGTACCAAACCATTCA	1	141	88.41
WSµ23^a^	F-TTTTGGTGGGATTCATA R-ATAAAAGGGTTAGAAAGACT	5	130–146	89.91
WSµ24	F-GTAAGGCATGAGAGACTAAG R-GTGTGATTTAGGATTGTT	1	237	49.27

^a^Polymorphic loci of high amplification success rate (> 80%) which were used for genetic diversity analysis

### Data analysis

PCR products were sized with GeneMapper Software v4.0 (Applied Biosystems). GenAlEx v 6.5 [7, 8] was used for the estimation of allele frequencies, performing Chi square test for deviations from Hardy–Weinberg equilibrium (HWE), and estimating probability of identity (PI), diversity measures (observed and expected heterozygosities, and observed and effective number of alleles), and statistically significant AMOVA Fst, G’stH (Hedrick’s standardized Gst) and Dest (Jost’s estimate of differentiation) [9] for genetic structuring between temporally separated populations. Linkage disequilibrium (LD) between pair of loci was tested by GenePop web version 4.2 [10, 11] by 10,000 dememorisation 500 batches and 10,000 iterations per batch. Polymorphic information content (PIC) was calculated by Cervus version 3.0.7 [12]. An assignment test was performed manually to assign the unique genotypes to different populations.

### Results

Of the 145 feather samples collected, genetic data could be obtained for 109, since other samples failed to amplify. Of the 9 microsatellites, only WSµ09, WSµ13, WSµ17, WSµ18 and WSµ23 were polymorphic (Table 1). The amplification success rate of different loci was uneven in that while WSµ08, WSµ13 and WSµ24 exhibited low amplification success rates (< 50%), WSµ03, WSµ09, WSµ17, WSµ18, WSµ20 and WSµ23 exhibited comparatively higher amplification success rates (> 80) (Table 1). Only those polymorphic loci that showed high amplification success i.e. WSµ09, WSµ17, WSµ18 and WSµ23 were subjected to population genetic analysis. Since a highly significant LD was detected between WSµ17 and WSµ18 (P = 0.0000, across all populations; according to Fisher’s method) and WSµ17 was not in HWE in any of the nesting seasons and showed lower amplification success and PIC-value than WSµ18 (Table 2), WSµ17 was dropped from further analysis.

**Table 2:** Diversity statistics of microsatellites for Delhi Zoo Painted Stork populations across three nesting seasons (2011–2014)

Nesting season	Locus	Missing data frequency	PIC	HWE P value	PI	N	Na	Ne	Ho	He	UHe	F
2011–2012	WSµ09	0.091	0.185	0.469	0.652	30	2.000	1.260	0.233	0.206	0.210	− 0.132
	WSµ17	0.182	0.197	0.000	–	–	–	–	–	–	–	–
	WSµ18	0.121	0.288	0.170	0.500	29	4.000	1.436	0.310	0.304	0.309	− 0.022
	WSµ23	0.121	0.624	0.130	0.159	29	4.000	3.132	0.621	0.681	0.693	0.088
Overall PI/mean ± SE					0.052	29.333 ± 0.333	3.333 ± 0.667	1.943 ± 0.597	0.388 ± 0.118	0.397 ± 0.145	0.404 ± 0.147	− 0.022 ± 0.064
2012–2013	WSµ09	0.056	0.274	0.102	0.506	34	2.000	1.486	0.235	0.327	0.332	0.280
	WSµ17	0.194	0.257	0.006	–	–	–	–	–	–	–	–
	WSµ18	0.139	0.264	0.766	0.533	31	3.000	1.395	0.323	0.283	0.288	− 0.140
	WSµ23	0.083	0.651	0.624	0.136	33	5.000	3.265	0.727	0.694	0.704	− 0.048
Overall PI/mean ± SE					0.037	32.667 ± 0.882	3.333 ± 0.882	2.049 ± 0.609	0.428 ± 0.152	0.435 ± 0.130	0.441 ± 0.132	0.031 ± 0.128
2013–2014	WSµ09	0.125	0.300	0.390	0.467	35	2.000	1.582	0.314	0.368	0.373	0.145
	WSµ17	0.200	0.290	0.003	–	–	–	–	–	–	–	–
	WSµ18	0.150	0.333	0.302	0.438	34	4.000	1.546	0.353	0.353	0.359	0.001
	WSµ23	0.100	0.650	0.191	0.140	36	5.000	3.345	0.833	0.701	0.711	− 0.189
Overall PI/mean ± SE					0.029	35.000 ± 0.577	3.667 ± 0.882	2.158 ± 0.594	0.500 ± 0.167	0.474 ± 0.114	0.481 ± 0.115	− 0.014 ± 0.097
Mean ± SE over all populations						32.333 ± 0.882	3.444 ± 0.412	2.050 ± 0.302	0.439 ± 0.075	0.435 ± 0.066	0.442 ± 0.067	− 0.002 ± 0.050
All seasons combined as a single population	WSµ09	0.092	0.262	0.127	0.524	99	2.000	1.450	0.263	0.310	0.312	0.153
	WSµ17	0.193	0.254	0.000	–	–	–	–	–	–	–	–
	WSµ18	0.138	0.300	0.105	0.484	94	4.000	1.463	0.330	0.317	0.318	− 0.041
	WSµ23	0.101	0.655	0.067	0.136	98	5.000	3.355	0.735	0.702	0.706	− 0.047
Overall PI/mean ± SE					0.034	97.000 ± 1.528	3.667 ± 0.882	2.089 ± 0.633	0.442 ± 0.147	0.443 ± 0.130	0.445 ± 0.130	0.022 ± 0.066

*PIC* polymorphic information content, *HWE P value* Hardy–Weinberg equilibrium P value, *PI* probability of identity, *N* sample size, *Na* observed number of alleles, *Ne* effective number of alleles, *Ho* observed heterozygosity, *He* expected heterozygosity, *uHe* unbiased expected heterozygosity, *F* fixation index, *SE* standard error

Diversity measures from different loci are given in Table 2. The mean observed and effective number of alleles range from 3.333 ± 0.667 (2011–2012) to 3.667 ± 0.882 (2013–2014), and from 1.943 ± 0.597 (2011–2012) to 2.158 ± 0.594 (2013-–2014), respectively. The mean observed and expected heterozygosities (H_o_ and He) range from 0.388 ± 0.118 (2011–2012) to 0.500 ± 0.167 (2013–2014) with mean = 0.439 ± 0.075 and from 0.397 ± 0.145 (2011–2012) to 0.474 ± 0.114 (2013–2014) with mean = 0.435 ± 0.066, respectively. In combined single population the H_o_, H_e_ and uH_e_ are 0.442 ± 0.147, 0.443 ± 0.130 and 0.445 ± 0.130, respectively.

The PI of multilocus genotypes for different breeding seasons is given in Table 2. The multilocus PI value of data (0.034) is much higher than the logical cut-off point value (0.0092) (the reciprocal of sample size, 109) i.e. the level of PI below which we have some confidence that two similar genotypes are likely to originate from the same individual. However, 42 unique genotypes were identified, 27 of which were represented by single samples, while the remaining 15 were represented by multiple samples. From 42 unique genotypes, 9, 7 and 13 genotypes were unique for nesting season 2011–2012, 2012–2012 and 2013–2014 respectively. Pairwise 8, 5 and 8 genotypes were shared between years 2011–2012 and 2012–2013, 2011–2012 and 2013–2014, 2012–2013 and 2013–2014, respectively, while 4 genotypes were common in all 3 nesting seasons. Based on the assignment test, 13, 15 and 14 unique genotypes were assigned to nesting season 2011–2012, 2012–2013 and 2013–2014, respectively. Interestingly, some unique genotypes of a particular year were assigned to other years also. For instance, from the 9 genotypes which were unique to year 2011–2012, 3 each were assigned to 2012–2013 and 2013–2014. Similarly, from the 13 genotypes which were unique to 2013–2014, 3 were assigned to 2011–2012 and 8 were assigned to 2012–2013.

Lower and non-significant estimates of traditional AMOVA Fst (Fst = 0.003, P = 0.230) and some new replacement statistics i.e. Hedrick’s standardized Gst (G’stH = 0.005, P = 0.247) and Jost’s D (Dest = 0.003, P = 0.250) indicate a lack of genetic structuring in three temporally distributed populations. Even when the tests were performed with only unique genotypes of each season, the non-significant values of Fst (Fst = 0.003, P = 0.315), Hedrick’s standardized Gst (G’stH = 0.004, P = 0.386) and Jost’s D (Dest = 0.002, P = 0.386) indicate lack of population structuring.

### Discussion

The nature and size of cross-amplified loci can vary among the source and the target species. Many of the WS polymorphic microsatellites are reported to be monomorphic in Jabiru Stork (JS) (*Jabiru mycteria*) [13]. While WSµ03 and WSµ20 are monomorphic in PS, in JS and European White Stork (EWS) (*Ciconia ciconia*) they are reported to be polymorphic [13, 14]. Loci WSµ08 and WSµ24, which are polymorphic in WS, are monomorphic in both PS and JS [13]. DNA replication slippage is considered as a major source of microsatellite variability [15]. Except for WSµ17, variations in allele sizes of polymorphic loci of PS are in agreement with the size of repeat motifs described in WS [6]. WSµ17 was described as a tetranucleotide in WS, but in PS we also observed an allele which varies in length from others by 2 bp. Although WSµ08 shows two alleles of size difference of around 1 bp, we counted it as monomorphic because in WS it was described as dinucleotide marker [6]. Such size differences could be because of mutations outside the stretch of repeat strand. WSµ09, WSµ13, WSµ18 and WSµ23 were as expected tri-, di-, tetra- and tetranucleotide microsatellites, respectively. The number of alleles over four polymorphic loci in PS is more than the number reported over same loci in WS.

Although all of the reported WS microsatellites are known for 100% cross-amplification success in congeneric PS and Milky Stork (*Mycteria cinerea*) [3, 16], the reasons of high amplification failures of some loci could be explained in terms of general problems of insufficient and degraded templates from non-invasive samples [3, 17] or in terms of possibility of null alleles due to mutations in primer binding sites in certain lines [18]. Due to the high probability of amplification failure because of the poor DNA quantity and quality from non-invasive samples, the possibility of null alleles could not be tested. Even for the samples which failed to amplify the loci on first attempt, the PCR experiment was performed repeatedly (3–4 times) with minute changes in the concentrations of the reagents and cycling conditions. In some cases the amplification success was seen on repeated PCR experiments. Five samples were repeatedly genotyped for 3 times and each time the similar fragment size was reported, which indicated a 0% scoring error. Maximum missing data for WSµ17 could be the reason for its significant deviations from HWE. LD between WSµ17 and WSµ18 was also reported in WS [6] and EWS [14], which indicates that in stork species these two loci are closely linked.

In WS, most of the studies report low-moderate genetic diversity in colonies of Americas [6, 19, 20], which are comparable with the 3 microsatellite limited genetic diversity encountered in PS of Delhi Zoo. As per the general trend of genetic diversity for waterbirds [21], low-moderate genetic diversity in storks could be because of being inhabitants of wetland habitat and their larger body mass. Some other factors have also been attributed to low genetic diversity in other storks. For instance, in the case of EWS it has been attributed to historical bottlenecks [14].

Field and molecular studies in WS colonies have indicated a lack of differentiation and low natal site philopatry [19, 20, 22–27]. Although the assignment test and differentiation results of our study indicate a lack of genetic structuring, due to low number of loci used, the presence of similar genotypes between the nesting seasons is not a strong reason to conclude that it is indeed the same individuals which are visiting the zoo year after year. Field observations indicate that in any given season PS can fluctuate between the short distance separated colonies [28]. So to be more certain if PS display nesting site fidelity, there is a need to test the genetic connectivity of multiple, spatially and temporally distributed PS colonies. Any high degree of fidelity in PS of Delhi Zoo, if indeed such is the case, may be on account of the fact that the zoo premises continue to offer a relatively safe environment for nesting [1].

Being an exclusively piscivorous bird [1], PS is a flagship of wetland ecosystems which are gravely threatened all across the Indian subcontinent. Its wide geographical spread and capacity to disperse far away from natal sites warrants further studies along biogeographical lines. Molecular genetic studies are likely to be extremely relevant in this regard, besides providing important baseline data for conservation of this species [2].

## Limitations of study

The limitations of the present study are the exclusive use of non-invasive samples and low number of useful polymorphic microsatellites. The fact that around 25% samples showed high PCR failures at most of the loci, severely limited our study. Except for some initial studies [19, 20], the number of microsatellites used in most of the genetic diversity studies on WS are considerably larger [29] than what we have managed. Higher number of polymorphic loci results an increase in confidence that similar genotypes are from same individual. For further improvements, we recognize the need to develop species specific markers of PS.

## Abbreviations

PS: Painted Stork
WS: Wood Stork
PI: probability of identity
PIC: polymorphic information content
LD: linkage disequilibrium
HWE: Hardy–Weinberg equilibrium
BSA: Bovine serum albumin
PCR: polymerase chain reaction
JS: Jabiru Stork
EWS: European White Stork
MS: Milky Stork
